# Does HOXA9 Gene Expression in Egyptian Chronic Myelogenous Leukemia Patients Affect Disease Progression? A Retrospective Cohort Study

**DOI:** 10.4274/Tjh.2012.0083

**Published:** 2013-12-05

**Authors:** Manar Mohamd Mohamad Ismail, Moneer M. Manar

**Affiliations:** 1 Laboratory Medicine Department, Faculty of Applied Medical Science, Um Al Qura University, Saudi Arabia; 2 Epidemiology and Biostatistics Department, National Cancer Institute, Cairo University, Egypt

**Keywords:** Chronic myeloid leukemia, CML, Accelerated phase HOXA9 gene, BCR-ABL expression, BCR-ABL/ABL ratio

## Abstract

**Objective:** Chronic myelogenous leukemia (CML) is a clonal stem cell disease and is consistently associated with the BCR-ABL fusion gene. The chronic phase of the disease tends to pass into an accelerated phase and eventually leads to acute leukemia if left untreated. Oncoproteins necessary for leukemic transformation are both fundamentally and clinically relevant to identify as they might be new molecular targets for the development of specific anti-leukemic drugs. This study is an initial step to define the proportion of HOXA9 gene expression in some Egyptians with chronic-phase CML at diagnosis and to evaluate its relation with BCR-ABL expression and its clinical significance.

**Materials and Methods:** Sixty-two newly diagnosed CML patients (56 in chronic phase, 1 in accelerated phase, and 5 in blastic crises) were enrolled in the study. HOXA9 and BCR-ABL gene expressions were detected by one-step RT-PCR. ABL was chosen as a control gene to calculate HOXA9/ABL and BCR-ABL/ABL ratios from densitometric values of PCR product intensities.

**Results:** HOXA9 expression was encountered in 25/56 (44.6%) of newly diagnosed CML patients in the chronic phase. The median expression was 0.31 (range: 0.08-1.37) in relation to the ABL gene, with a higher frequency of expression in CML patients presenting with splenomegaly (p<0.001), high Sokal score (p<0.001), and BCR-ABL expression from the first round (p=0.004). No association could be detected with other clinical parameters, overall survival, or disease-free survival.

**Conclusion:** HOXA9 expression is closely related to poor prognostic factors, but we could not demonstrate its relationship to patient survival.

**Conflict of interest:**None declared.

## Abstract (tr)

**Amaç:** Kronik Miyeloid Lösemi (KML) klonal bir kök hücre hastalığıdır ve BCR-ABL füzyon geni ile ilişkilidir. Hastalık tedavi edilmediği zaman, kronik evreden hızlanmış evreye ilerler ve sonunda akut lösemi ile sonuçlanır. Lösemik transformasyonda temel olarak gerekli olan ve klinik olarak ilişkili onkoproteinlerin belirlenmesi spesifik anti-lösemik ilaçlar için yeni moleküler hedef olabilecekleri için önemlidir. Bu çalışma bazı Mısırlı kronik evre KML hastalarında HOXA9 gen sunum oranını belirlemede ve bunun BCR-ABL sunumu ile ilişkisinin ve klinik öneminin değerlendirilmesinde başlangıç adımıdır.

**Gereç ve Yöntemler:** Çalışmaya altmış iki yeni tanı KML olgusu (56 kronik evre, 1 hızlanmış evre ve 5 blastik kriz) alındı. HOXA9 ve BCR-ABL genlerinin sunumu tek basamaklı RT-PCR ile tespit edildi. HOXA9/ABL ve BCR-ABL/ABL oranlarının PCR ürün yoğunluklarının dansitometrik değerleri üzerinden hesaplanması için kontrol geni olarak ABL seçildi.

**Bulgular:** HOXA9 sunumu yeni tanı kronik evre KML olgularının %44.6’sında (25/56) tespit edildi. ABL geni ile ilişkili olarak ortanca sunum 0.32 idi (aralık: 0.08-1.37) ve splenomegali ile başvuran (p<0.001), yüksek Sokal skoru (p<0.001) ve birinci raundda BCR-ABL sunumu olan (p =0.004) KML olgularında daha yüksek sunum sıklığı vardı. Diğer klinik parametreler, genel sağkalım ve hastalıksız sağkalım ile ilişki tespit edilemedi.

**Sonuç:** HOXA9 sunumu kötü prognostik faktörler ile yakından ilişkilidir ancak çalışmamızda bunun hasta sağkalımı ile ilişkisini gösteremedik.

## INTRODUCTION

Chronic myelogenous leukemia (CML) has a worldwide annual incidence of 1-2 cases per 100,000. It can occur at any age, but the median age at diagnosis is 40-59 years [1]. CML is a clonal stem cell disease and is consistently associated with the BCR-ABL fusion gene located on the Philadelphia chromosome [2]. The translocation fuses the BCR and ABL genes, which results in the production of oncoprotein with an aberrant tyrosine kinase, which confers proliferative and survival properties to hematopoietic cells [3].This kinase plays a critical role in the pathogenesis of CML by activating multiple signaling pathways such as Ras, PI3K, MAPK, JAK/STAT, and Myc [4]. In the early phases of the disease there is excessive accumulation of mature myeloid cells that pass into the accelerated phase and eventually develop to acute leukemia if left untreated [1]. Additional genetic changes may reflect genetic instability. Therefore, intrinsic aggressiveness of the disease has been reported to ensue at varying frequencies during disease progression to the accelerated and blast crisis phases [5,6]. 

The genetic events involved in CML’s transformation into the acute phase are poorly understood [7]. However, there is increasing evidence that abnormal HOXA protein expression is functionally significant in myeloid transformation [8]. The homeodomain protein of the HOX family plays an important role in regulating definitive hematopoiesis [9]. One of them, HOXA9, part of the A cluster on chromosome 7p15, is expressed under physiological conditions in primitive hematopoietic cells of human and murine origin. The expression pattern of the homeobox genes in hematopoietic cells is specific to both lineage and differentiation stage. This expression is down-regulated as blood cells differentiate, suggesting a function in early hematopoiesis [10]. 

A growing body of evidence supports the notion that misexpression of the HOXA9 homeobox gene is a common and critical event in human acute myelogenous leukemia (AML) and is critical to the induction and maintenance of the malignant phenotype [9,11]. It was also proven that enforced expression of HOXA9 in murine marrow cells can immortalize the cells in culture and thus contributes largely with other events in leukemogenesis [12]. 

The strong association between HOXA9 overexpression and development of AML has encouraged us to determine its expression in CML at diagnosis to determine its proportion among Egyptian patients and to evaluate its relation with BCR-ABL expression and the clinical significance of such expression in disease aggression and patient survival. 

## MATERIALS AND METHODS

**Study Design**

**Patients and Clinical**

Samples Peripheral EDTA blood samples (5 mL) were obtained from 62 new patients presenting to the outpatient clinic of the National Cancer Institute, Cairo University, during a 6-month period starting in March 2004 with suspected CML based on morphological examination of peripheral blood (PB) and bone marrow (BM) films and leukocyte alkaline phosphatase score. Diagnosis was confirmed by the presence of the BCR-ABL fusion gene either from the first round or by nested polymerase chain reaction (PCR). Fifty-six patients were in the chronic phase, 1 was in the accelerated phase, and 5 had acute blastic crises (ABCs) on top of CML (2 with B-cell acute lymphocytic leukemia [B-ALL] and 3 with AML) according to World Health Organization classifications [13]. The Sokal score, a prognostic score that depends on age, spleen size, PB blasts, and platelets [14], was calculated. Overall survival and disease-free survival (DFS) were calculated for all patients and in relation to the studied genes. The study was approved by the local ethics committee of the university. All patients presenting in the chronic phase were treated with hydroxyurea at 1 to 6 g/day orally, depending on the level of the white blood cell (WBC) count [15]. When the total leukocyte count (TLC) reached 20x109/L, the dosage was decreased to 1 to 2 g/day and given continuously with the goal of reaching normal WBC counts (5 to 15x109/L).The drug was temporarily discontinued if the WBC count dropped below 5x109/L [16]. 

**RNA Purification**

Total RNA was extracted from 106 cells from PB EDTA samples using the QIAamp RNA Blood Mini Kit (QIAGEN, Cat. No. 52304) and stored at -80 °C. 

**RT-PCR**

The OneStep RT-PCR kit (QIAGEN, Cat. No. 210212), which combines cDNA synthesis from RNA with PCR amplification to provide a rapid, sensitive method for analyzing gene expression, was used. The following primer sets were used: 

TGTGGTTCTCCTCCAGTTGATAGA/TCGGTGAGGTTGAGCAGTCGAG, which amplifies a fragment of 267 bp for human HOXA9 [9]; 

TGTTGACTGGCGTGATGTAGTTGCTTGG/TCAGCGGCCAGTAGCATCTGACTT for ABL, which was used as an internal control; 

ACAGCATTCCGCTGACCATCAATAAG/TGTTGACTGGCGTGATGTAGTTGCTTGG (BCR-ABL, first round); and CTGACCATCAATAAGGAAG/GACCCGGAGCTTTTCACCTTTAGTT (BCR-ABL; second round) [17]. 

The total reaction volume was 25 µL, containing 2.5 µL of RNA, 100 µM of each dNTP, 0.4 mM of each primer (forward and reverse primer for each gene), and the enzyme mix included in the kit (reverse transcriptase and hot-start Taq DNA polymerase) in a 1X RT reaction buffer. All RT-PCR reactions included NTC control (reaction mix without RNA). The confirmation of BCR-ABL amplification was carried out by nested PCR if the sample did not reveal it from the first round. 

**Cycling Parameters**

The thermal cycle program included a step for reverse transcription (30 min, 50 °C); an initial PCR activation step (15 min, 95 °C); 30 cycles consisting of denaturation (1 min, 94 °C), annealing (1 min, 58 °C), and extension (1 min, 72 °C); and a final extension step (10 min, 72 °C). 

**Electrophoresis**

**Figure 1:**
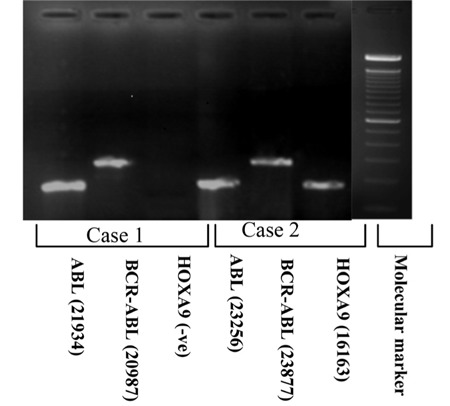
Agarose gel electrophoresis for PCR product. Case 1: A case of CML that failed to express the HOXA9 gene at 276 bp. Case 2: Another case of CML that did express the HOXA9 gene. Molecular size marker: 100-1000 bp. Band density is presented between brackets.

Ten microliters of the PCR products were subjected to electrophoresis on 2% agarose gel containing ethidium bromide. A molecular weight marker (100-1000 bp) was used to assess the positions of the defined DNA band. The gels were visualized under UV light (Figure 1).The image obtained was analyzed using complete gel documentations and an analysis system (Biometra, Germany). In order to obtain a semi-quantitative value, the intensity of the gene of interest (HOXA9 or BCR-ABL) was compared to a control gene in the same sample [18]. ABL was chosen as a control gene [19]. The HOXA9/ABL and BCR-ABL/ABL ratios were calculated from densitometric values of PCR product intensities. 

**Statistical Methods**

Data were analyzed using SPSS 12. The chi-square test (Fisher’s exact test) was used to examine the relation between qualitative variables. Spearman’s rho method was used to test correlations between numerical variables. The Kaplan–Meier method was used for survival analysis with the log-rank test to compare survival curves. All tests were 2-tailed and p<0.05 was considered significant. 

## RESULTS

The clinical characteristics of chronic-phase CML patients are shown in Table 1. HOXA9 expression was encountered in 25/56 (44.6%) of newly diagnosed CML cases. The median expression was 0.31 (range: 0.08-1.37) in relation to the ABL gene in each sample. The expression of HOXA9/ABL ratio in the accelerated case was 0.31, and in the 3 myeloid blastic crisis cases it was 0.83, 0.59, and 0.51, while it was not expressed in cases of lymphoid crisis. 

HOXA9 was not related to age, sex, percentage of blasts in PB or BM, hemoglobin levels, or platelet count (p>0.05). HOXA9-positive CML was significantly associated with larger spleen size (15.9±2.5 cm vs. 5.4±3.2 cm, p<0.001), higher Sokal score (p<0.001), and BCR-ABL expression from the first round (p=0.004) (Table 2). The HOXA9/ABL ratio was positively correlated with the BCR/ABL ratio (r=0.538, p=0.008), but not correlated to Sokal score (r=0.001, p=0.995). 

**Survival Analysis**

**Figure 2:**
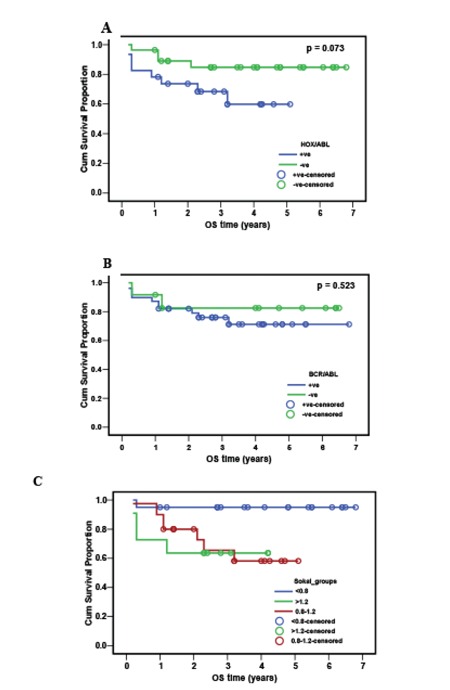
Overall survival in 56 chronic-phase CML patients. A) HOXA9/ABL ratio in relation to overall survival (OS). B) BCR/ABL ratio in relation to OS. C) Sokal score in relation to OS.

The median follow-up for the chronic-phase CML patients was 3 years (range: 0.2-6.8). The cumulative overall survival was 77.5%. There was no significant relation between overall survival and expression of the HOXA9 gene (p=0.073) or BCR-ABL fusion gene expression whether from the first round or the second round (p=0.523). Within the HOXA9-positive cases, there was no significant relation between HOXA9/ABL ratio and overall survival (p=0.794). Patients with a Sokal score of <0.8 had significantly higher overall survival (95%) compared to the other 2 groups with higher scores (p=0.017 and p=0.022) (Table 3; Figure 2). 

Ten out of 56 patients progressed to either the accelerated phase or acute blastic crisis (5 progressed to acute leukemia and the other 5 to the accelerated phase), and 5 cases could not be followed. Regarding the patients that progressed to acute leukemia, the 3 that developed AML had HOXA9/ABL ratios of 0.31, 0.47, and 1.37 at diagnosis, while the other 2 who developed ALL did not express HOXA9. Regarding the accelerated cases, only 1 patient had an HOXA9/ABL ratio of 0.25 at presentation. Considering Sokal scores, 3 patients passed to the accelerated phase and 1 developed ABC in the group with low scores (<0.8), 2 patients progressed to the accelerated phase and 3 developed ABC in the group with intermediate scores (0.8-1.2), and only 1 developed ABC in the high score group (>1.2). 

**Figure 3:**
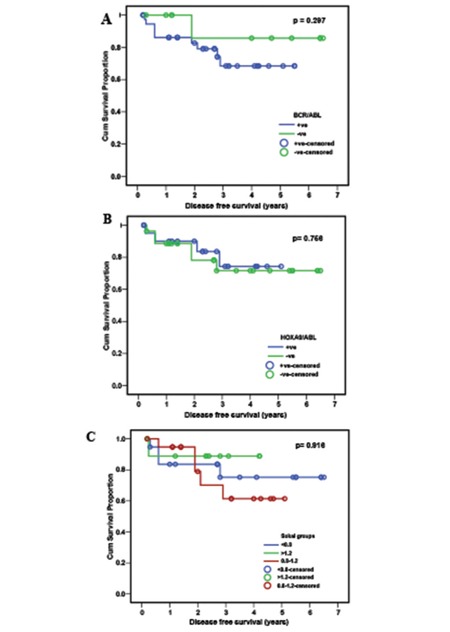
Disease-free survival (DFS) in 56 chronic-phase CML patients. A) HOXA9/ABL ratio in relation to DFS.B) BCR/ABL ratio in relation to DFS. C) Sokal score in relation to DFS.

The cumulative DFS for those who did not express HOXA9 was 71.7% versus 73.4% among HOXA9-positive cases (p=0.759). Within the HOXA9-positive cases, there was no significant relation between DFS and HOXA9/ABL ratio (p=0.337). DFS was 68.4% for cases in which BCR-ABL was expressed from the first round versus 85.7% for cases in which it was expressed from the second round (p=0.297). DFS was 75.2% in cases with a Sokal score of <0.8, 61.4% for score of 0.8-1.2, and 88.9% for score of >1.2, with no significant difference among the 3 levels (p>0.05) (Figure 3).

## DISCUSSION

This study demonstrated an HOXA9 expression rate of 44.6% in patients with chronic-phase CML. A previous study found HOXA9 expressed at detectable levels in every sample [20]. Our results could be explained by the fact that the expression of HOXA9 is down-regulated during myeloid differentiation [21], and all of the cells in chronic-phase CML show myeloid differentiation. 

In accordance with other studies, we found lower expression of the HOXA9/ABL ratio in the accelerated cases than in cases of myeloid blastic crisis [22,24], raising the possibility that HOXA9 may interact with BCR-ABL to transform BM cells. 

In the current series, patients presenting with lymphoid crises failed to express HOXA9. This could be explained by the fact that over-expression of HOXA9 in more mature cells enhances granulopoiesis and partially blocks B lymphopoiesis [12]; thus, it would not be expressed in B-ALL. In addition, in a previous gene expression study of human leukemia, HOXA9 emerged as one of the top 20 genes that distinguished AML from ALL [25]. 

In the current study, patients with poor prognosis (i.e. intermediate or high Sokal score) demonstrated higher HOXA9 expression (p<0.001), which concurs with the results of previous studies [20,26]. Splenomegaly was associated with HOXA9 expression (p<0.001), which is one of the factors included in Sokal scores denoting poor prognosis. Splenomegaly was also a criterion found in an experimental animal study done by Mayotte et al., in which they induced leukemia by HOXA9 over-expression [24]. 

In this work, 92% of HOXA9-positive cases had BCR-ABL expressed from the first round (p=0.004), i.e. patients with more copies of the BCR-ABL fusion gene showed higher proportions of HOXA9 expression. A previous study reported that patients with poor prognosis had increased expression of BCR-ABL as well as the HOXA9 gene [20]. 

In the current study, overall survival was 77.5% without significant relation to expression of the HOXA9 gene (p=0.073) or BCR-ABL (p=0.523). Overall survival was 95% for cases with a Sokal score of <0.8, which is significantly higher as compared to the other 2 groups (p=0.017 and p=0.022). 

DFS was not significantly related to HOXA9 or BCR-ABL expression or to Sokal score (p>0.05). Contrary to these findings, a previous study reported a patient with poorer prognosis (high Sokal score) showing the highest HOXA9/ABL ratio, who quickly entered blast crisis and died 5 months later [26]. 

In this cohort study, 5 patients progressed to acute leukemia; of those, 3/5 expressed HOXA9 at diagnosis and developed AML, while the other 2, who developed ALL, did not express HOXA9. These data support previously recorded results of an earlier experimental study in which all mice that received BM cells infected with BCR-ABL plus HOXA9 retroviruses died within 9 days of acute leukemia and, in all cases, the leukemia was myeloid [24]. The fact that the patients presenting with AML on top of CML in this study were expressing higher levels of HOXA9/ABL ratio may indicate that the combination of these oncogenes was sufficient for the full transformation into AML. 

## CONCLUSION

The rate of HOXA9 expression in the studied chronic-phase CML cases was 44.6%. It was higher in cases of poor prognosis with high or intermediate Sokal scores and in patients that expressed the BCR-ABL fusion gene from the first round. We could not draw a firm conclusion about whether HOXA9 expression has a bad effect on overall or disease-free survival. However, for data regarding the proportion of HOXA9 expression in CML and its effect on blastic transformation, HOXA9 should be evaluated in a larger number of patients both at presentation and during blastic crisis, and it will be important to evaluate misexpression of HOXA9 oncogenes when seeking genes involved in the progression of CML to acute myeloid leukemia. 

**Authors’ Contributions**

Manar Ismail was responsible for study design, all lab work, collection of clinical data, analysis and interpretation of findings, and writing of the paper. 

Manar Moneer was responsible for statistical analysis, interpretation of the data, and revising of the paper. 

**Acknowledgments**

The authors acknowledge Dr. Heba Shaker for her scientific support, expert technical assistance, and permission to perform the practical work under her supervision in the BMT lab at the NCI, Cairo University. 

## CONFLICT OF INTEREST STATEMENT

The authors of this paper have no conflicts of interest, including specific financial interests, relationships, and/ or affiliations relevant to the subject matter or materials included. 

## Figures and Tables

**Table 1:**
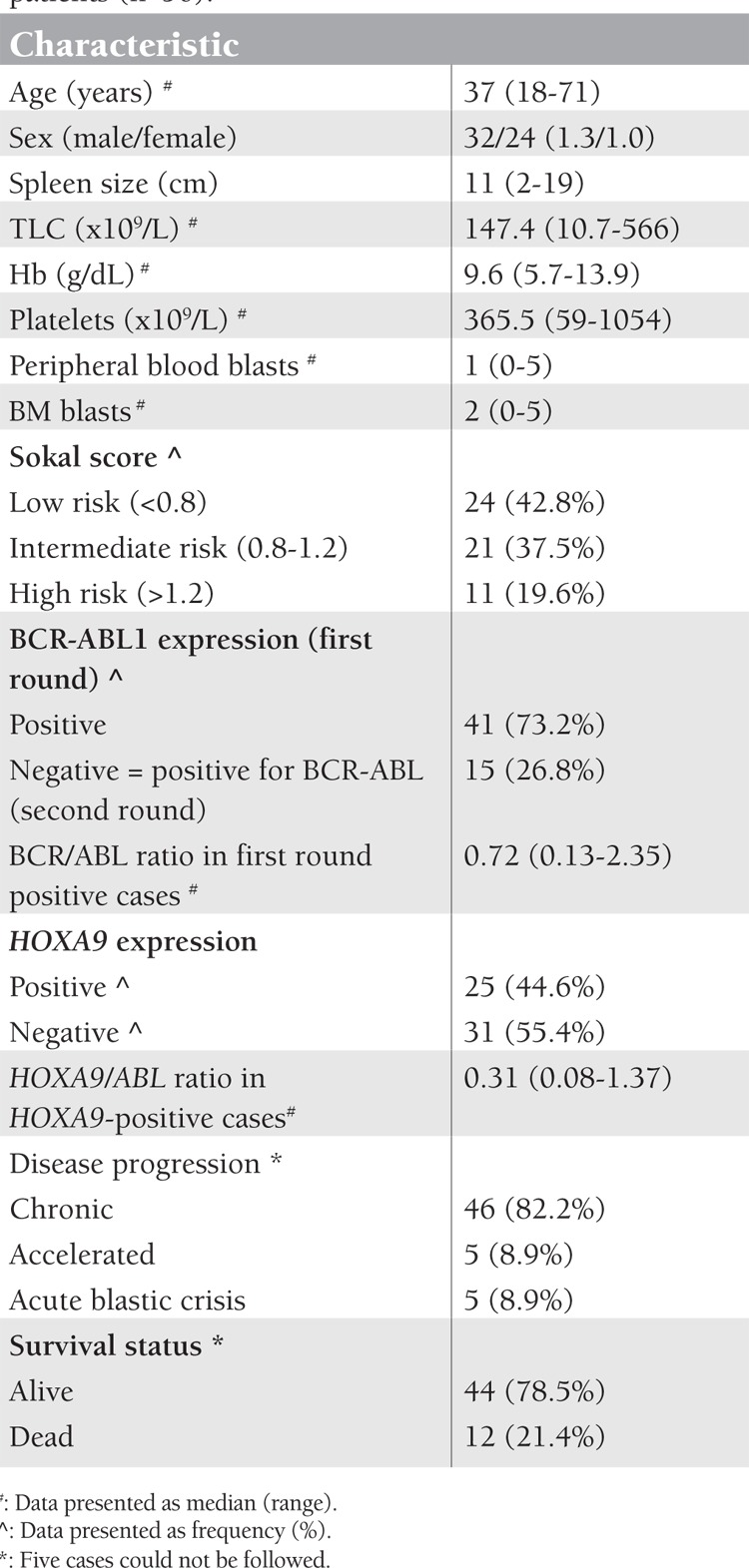
Clinical characteristics of chronic-phase CML patients (n=56).

**Table 2:**
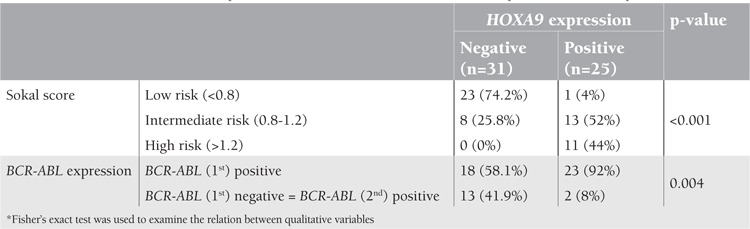
Relation between HOXA9 expression and both Sokal score and BCR-ABL expression in chronic-phase CML

**Table 3:**
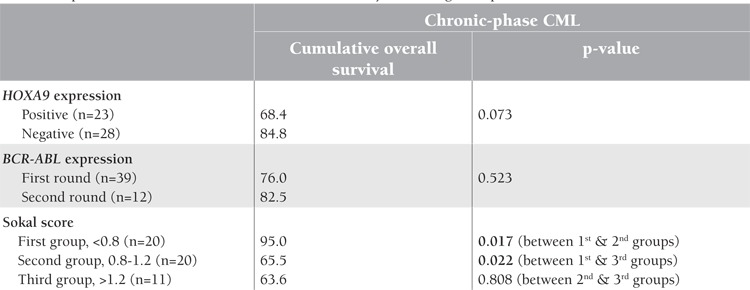
Impact of the studied factors on overall survival at 3 years among CML patients.
